# Cortical Tubers’ Transformation in Pediatric Patients Diagnosed with Tuberous Sclerosis Complex: A Retrospective Longitudinal MRI Analysis

**DOI:** 10.3390/jcm14217665

**Published:** 2025-10-29

**Authors:** Camilla Russo, Simone Coluccino, Maria Fulvia De Leva, Stefania Graziano, Carmela Russo, Federica Mazio, Maria De Liso, Domenico Cicala, Anna Nastro, Federica Palladino, Serena Troisi, Pietro Spennato, Giuseppe Cinalli, Antonio Varone, Eugenio Maria Covelli

**Affiliations:** 1 Neuroradiology Unit, Department of Neurosciences, Santobono-Pausilipon Children’s Hospital, AORN, 80129 Naples, Italy; camilla_russo@hotmail.it (C.R.); s.coluccino@santobonopausilipon.it (S.C.); russocarmela84@gmail.com (C.R.); federicamazio1@gmail.com (F.M.); maria.delisomd@gmail.com (M.D.L.); domenico.cicala@gmail.com (D.C.); anna_nastro@libero.it (A.N.); 2 Neurology Unit, Department of Neurosciences, Santobono-Pausilipon Children’s Hospital, AORN, 80129 Naples, Italy; m.deleva@santobonopausilipon.it (M.F.D.L.); s.graziano@santobonopausilipon.it (S.G.); f.palladino@santobonopausilipon.it (F.P.); s.troisi@santobonopausilipon.it (S.T.); a.varone@santobonopausilipon.it (A.V.); 3 Neurosurgery Unit, Department of Neurosciences, Santobono-Pausilipon Children’s Hospital, AORN, 80129 Naples, Italy; p.spennato@santobonopausilipon.it (P.S.); giuseppe.cinalli@gmail.com (G.C.)

**Keywords:** tuberous sclerosis complex, cortical tuber, magnetic resonance imaging, follow-up

## Abstract

**Background**: Cortical tubers (CTs) are hallmark brain lesions in tuberous sclerosis complex (TSC), historically considered stable in number over time; prior literature has correlated overall CT burden on magnetic resonance imaging (MRI) with disease severity. As longitudinal imaging studies assessing CTs’ evolution over time are lacking, we aim to investigate temporal changes in CTs—both in number and signal—on MRI in a cohort of pediatric TSC patients. **Methods**: A retrospective single-center analysis was conducted on 57 pediatric TSC patients who underwent longitudinal MRI studies in a 10-year span. Required MRI sequences included volumetric unenhanced T1-weighted, SWI, T2w and/or FLAIR. CTs were evaluated by two neuroradiologists and classified into five subtypes (A, B, C1, C2, D) according to signal characteristics. Statistical comparison was performed using *t*-tests. **Results**: Paired *t*-test analysis demonstrated a significant longitudinal increase in the overall number of CTs, rising from 16.11 ± 12.43 at baseline to 18.77 ± 13.29 at follow-up (mean difference = −2.67, 95% CI [−3.94, −1.39]; t (56) = 4.19; *p* < 0.0001), corresponding to a moderate effect size (Cohen’s d ≈ 0.56). When stratified by age, patients <2 years—representing the incompletely myelinated subgroup—showed a more pronounced increase in CT burden, from 19.46 ± 15.21 to 24.17 ± 15.75 (mean difference = −4.71, 95% CI [−7.37, −2.04]; t (23) = 3.65; *p* = 0.0013; d ≈ 0.75). In contrast, patients aged ≥2 years demonstrated a smaller but still significant increase, from 13.67 ± 9.45 to 14.85 ± 9.64 (mean difference = −1.18, 95% CI [−2.08, −0.28]; t (32) = 2.68; *p* = 0.0115; d ≈ 0.46). Direct comparison between the two subgroups using Welch’s two-sample *t*-test confirmed that the mean CT count in patients <2 years was significantly higher than in those ≥2 years (mean difference = 3.53 ± 1.36; t = 2.59; df = 28.4; *p* = 0.0075), with a large effect size (Cohen’s d ≈ 0.78). Type C1-C2 tubers evolved from pre-existing earlier-stage lesions, while most newly visible CTs over time were type A-B. Type D tubers remained rare and derived from earlier-stage CTs. **Conclusions**: Contrary to previous assumptions, CTs in pediatric TSC showed a tendency to increase in number and evolve in signal over time, thus challenging the notion of stability and suggesting dynamic behavior. Incomplete myelination in early infancy may impact MRI CTs detection by reducing contrast with surrounding brain tissue, potentially leading to their underestimation/misidentification.

## 1. Introduction

First described in the 19th century, tuberous sclerosis complex (TSC) is a multisystem disorder marked by the development of dysplastic lesions and neoplastic growths across various organs; later understood—through advances in genetics and neuroimaging—to result from mutations in the *TSC1* or *TSC2* genes leading to dysregulated mTOR signaling, its prevalence is estimated at 1:20,000 worldwide, with an increase in incidence over time due to refining of diagnostic criteria and implementation of diagnostic tools. Central nervous system (CNS) involvement occurs in nearly all affected individuals [1,2,3]. Among CNS manifestations, cortical tubers (CTs) stand out as a hallmark, representing the most prominent and diagnostically valuable neurological finding in TSC [4,5]. The International Tuberous Sclerosis Complex Consensus Group’s 2021 diagnostic update underscores the indispensable role of neuroimaging, emphasizing the presence of CTs as a major diagnostic criterion and guiding both initial assessment and longitudinal follow-up via comprehensive brain MRI, especially in pediatric patients [6]. Indeed, pediatric-onset TSC is characterized by higher phenotypical heterogenicity and variable neurological/neuropsychiatric morbidity [7]. Importantly, cerebral CT burden is not merely diagnostic, as scientific evidence suggests a negative association between higher tuber count with ontogeny of normal brain function, greater neurological severity (including more severe epilepsy), developmental impairment, and complex cognitive maturity [8,9,10,11,12,13,14]. Pathologically CTs, defined as glio-neural hamartomas extending from cortical gray matter to subcortical white matter, exhibit hallmark features such as disrupted cortical lamination, balloon or giant cells, astrocytic gliosis, and abnormal neuronal differentiation [15]. Detailed histopathological studies have elaborated these structural aberrations, while more recent work using molecular and imaging-correlative approaches has further refined our understanding of tuber heterogeneity, revealing variation in astroglial and microstructural composition that may underlie clinical variability [15,16,17,18]. Together, these findings cement CTs as both a diagnostic cornerstone and a potential biomarker of disease severity in TSC, justifying the need for high-resolution, longitudinal neuroimaging studies mainly relying on magnetic resonance imaging (MRI), that is widely recognized as the reference method to investigate CNS involvement in TSC [4].

A wide spectrum of CT subtypes has been described in the literature, with the most widely adopted classification proposed by Gallagher et al. in 2010 [19]. On MRI, CTs more frequently appear as focal thickening and expansion of the gyri, characterized by hyperintensity of cortical gray matter with associated subcortical flame-shaped white matter abnormalities on T2-weighted sequences (including fluid-attenuated inversion recovery—FLAIR), corresponding to cortical hypointensity and subcortical hyperintensity on T1-weighted imaging. In infants, however, because myelination of neural tissue is physiologically incomplete, CTs present with a distinct appearance [4]: they are usually only mildly hyperintense on T1-weighted imaging and hypointense on T2-weighted sequences relative to the surrounding unmyelinated tissue. This age-related signal pattern may result in underestimation of CT burden during the first years of life, until myelination is complete [4,5,6]. In some cases, vacuolization has also been reported [20]. Beyond conventional T1- and T2-weighted sequences, diffusion-weighted imaging (DWI) and corresponding apparent diffusion coefficient (ADC) maps usually demonstrate elevated ADC values within CTs compared with adjacent normal brain. Another frequent MRI finding is the presence of intra-tuberal calcifications, which may be microscopic or macroscopic, round or gyriform in shape, sporadically associated with cystic degeneration or mild contrast enhancement [4,19,20,21,22,23]. Calcified components, variably implicated in tuber epileptogenicity [4,21,22], are notably not included in the Gallagher classification, despite their clinical relevance. Intra-tuberal calcifications are generally considered stable, with only isolated reports documenting progressive calcification over time [23].

Although CTs have traditionally been regarded as structurally stable lesions that do not change in number or size beyond what is proportional to normal brain growth, recent evidence suggests otherwise. Microstructural changes within tuberal and peri-tuberal tissues have been observed with aging [24], and in some cases cerebellar lesions have shown dynamic evolution [25,26]. Additionally, cyst-like lesions have been reported to increase in number and volume [27], and isolated observations suggest that cortical tubers may appear to change over time due to progressive calcification [23,25]. Apparent increases in CTs burden may also be attributable to changes in contrast visibility or growth proportional to normal brain development [28]. To date, however, no study has systematically quantified the potential for cerebral CTs to increase in number over time, particularly during the earliest years of life and in pediatric patients at diagnosis. Based on this gap, this MRI-based retrospective study aimed to examine whether cerebral CTs in pediatric patients diagnosed with TSC show appreciable changes in signal characteristics or in overall number over time. The study specifically focused on whether purely intracortical CTs are more difficult to detect during infancy, when myelination is still incomplete, in a cohort of unrelated children diagnosed with TSC.

## 2. Materials and Methods

### 2.1. Patients Selection

This retrospective study evaluated demographic and neuroimaging data from a total of 108 patients with a confirmed diagnosis of TSC who were regularly followed at the Neurology Unit of Santobono-Pausilipon Children’s Hospital in Naples, Italy, between 2014 and 2024. To ensure methodological rigor and homogeneity of imaging data, only those patients who underwent both baseline and longitudinal follow-up MRI examinations within the Neuroradiology Unit of the same institution were included, provided that all acquisitions were performed using the same 1.5 Tesla MRI scanner (Ingenia Stream, Philips Medical Systems, Utrecht, The Netherlands); this choice was motivated by the need to avoid confounding variables introduced by heterogeneous acquisition protocols or by differences in field strength and hardware across scanners, which could have potentially affected the sensitivity in detecting subtle imaging findings such as calcifications or small cystic changes. No preliminary sample size calculation was performed because the study is retrospective and observational in nature. All available patient records meeting the above-mentioned inclusion criteria were included to maximize the dataset; consequently, the sample size was determined by the number of eligible cases rather than a priori statistical power considerations.

Exclusion criteria were (i) patients who were older than 16 years at the time of diagnosis, in order to restrict the cohort to the pediatric population, which is most vulnerable to early-onset epilepsy and developmental sequelae; (ii) patients for whom either radiological or genetic data were missing; and (iii) patients whose MRI examinations were incomplete or uninterpretable due to substantial artifacts, including severe motion or device-related interference; (iv) notion of preterm or extremely preterm birth. In patients who subsequently underwent surgical interventions for drug-resistant epilepsy, such as CTs resection or MRI-guided laser interstitial thermal therapy, the MRI performed for presurgical planning was considered as the last reference follow-up study, given its diagnostic completeness and clinical relevance.

After applying these inclusion/exclusion filters, a final cohort of 57 consecutive, unrelated pediatric patients fulfilling international diagnostic criteria [6] for TSC was retained for analysis. The gender distribution was slightly skewed toward females (male:female ratio 24:33, corresponding to 42% and 58%, respectively). Genetic characterization showed a predominance of TSC2 mutations (n = 25, 44%), followed by TSC1 mutations (n = 12, 21%), and a group of patients with not molecularly identified mutation (n = 20, 35%). The mean age at baseline or onset MRI was 4.9 ± 4.8 years, while the mean age at the last follow-up MRI was 8.9 ± 5.3 years. Patients underwent MRI examinations with a mean interval of 4.0 ± 2.7 years between the first and last scans. Of note, 24 of 57 patients (42%) demonstrated incomplete myelination at baseline MRI, consistent with their young age at diagnosis (<2 years), whereas only 3 patients (5%) still showed incomplete myelination at the last follow-up scan.

### 2.2. MRI Acquisition and Tubers’ Classification

The MRI protocol applied for this study was specifically designed to balance diagnostic completeness with patient tolerability, acknowledging the challenges posed by pediatric imaging and by the frequent need for sedation especially in younger patients. To be considered eligible for retrospective evaluation, MRI examinations had to fulfill the following minimum requirements: (i) unenhanced T1-weighted imaging (usually 3D acquisition, isotropic resolution), providing high-resolution anatomical reference and allowing for multiplanar reconstructions; (ii) unenhanced T2-weighted and/or FLAIR imaging (possibly 3D acquisition, isotropic resolution), depending on patient age and degree of myelination (i.e., in younger patients, where incomplete myelination may reduce the diagnostic performance of FLAIR, conventional T2-weighted sequences were prioritized); (iii) DWI with corresponding ADC maps, also useful for excluding alternative pathologies as well as to identify confounding lesions; (iv) susceptibility-weighted imaging (SWI), which was included as a key sequence for the detection of subtle calcifications or hemosiderin deposits, given its sensitivity to local magnetic susceptibility changes. When available, post-contrast volumetric T1-weighted images were also reviewed, although they were not considered mandatory for inclusion; these were primarily used in cases of diagnostic doubt, to exclude secondary processes such as infection, neoplasia, or other disease-related complications that could mimic CTs.

All imaging examinations were retrospectively reviewed by two board-certified neuroradiologists with at least 10 years of experience in pediatric neuroimaging. The analyses were performed in consensus to minimize inter-reader variability; observers were blinded both to clinical and radiological temporal data. The primary objective of the imaging evaluation was to systematically assess longitudinal changes in number, signal, and architecture of CTs. For this purpose, a novel MRI-based classification of CTs was developed, expanding upon previously established systems but introducing specific categories to account for calcifications and cystic changes. CTs were stratified into five main types:

- Tuber A: Isointense on volumetric T1-weighted sequences and subtly hyperintense on T2-weighted sequences, without mass effect, gyral distortion, or calcifications detectable on SWI.
- Tuber B: Hypointense on volumetric T1-weighted sequences and diffusely hyperintense on T2-weighted sequences, poorly defined borders, minimal mass effect, slight gyral distortion, but still no SWI calcifications.
- Tuber C1 (micro-calcified): Hypointense on volumetric T1-weighted and hyperintense on T2-weighted sequences, with the presence of subtle, non-confluent, pinpoint-like calcifications visible on SWI.
- Tuber C2 (macro-calcified): Hypointense on volumetric T1-weighted and hyperintense on T2-weighted sequences, with large, confluent, linear or curvilinear calcifications on SWI, corresponding to more advanced calcific deposition.
- Tuber D (cystic): Hypointense on volumetric T1-weighted and hyperintense on T2-weighted sequences, with a central cystic component or vacuolization, regardless of the presence or absence of calcifications.

This refined classification was designed to capture subtle radiological variations that might potentially correlate with clinical features such as seizure severity, treatment response, and disease progression, particularly highlighting the potential pathogenic relevance of intra-tuberal calcifications.

### 2.3. Statistical Analysis

Statistical analyses were performed with the dual aim of describing the distribution of tuber types in the cohort and investigating longitudinal changes over time. Descriptive statistics were initially used to summarize the incidence, prevalence, and distribution of the different tuber subtypes, with results reported as absolute numbers, means ± standard deviation (SD), and percentages. For longitudinal comparisons, changes in tuber count and signal characteristics between baseline and final follow-up MRI examinations were assessed; variations were expressed in terms of overall number of tubers per patient, percentage change, and mean ± SD variation. To determine the statistical significance of these changes, paired *t*-tests were performed, adopting a conventional threshold of *p* ≤ 0.05 to indicate statistical significance. Same analyses were also performed stratifying patients in two subgroups according to age and myelination, (<2 years and ≥2 years), in order to determine whether incomplete myelination in early infancy may impact on CTs detection and count. In particular between-group comparisons were performed using Welch’s *t*-test, which does not assume equality of variances. Effect sizes were estimated using Cohen’s d, and 95% confidence intervals were calculated for the mean differences. The robustness of the test was considered adequate given its tolerance to moderate violations of normality and equality of variances. Interobserver agreement between the two readers was also assessed using the Intraclass Correlation Coefficient (ICC) based on a two-way random-effects model with absolute agreement. ICC values were interpreted according to common criteria: <0.5 = poor, 0.5–0.75 = moderate, 0.75–0.9 = good, and >0.9 = excellent reliability. All statistics were conducted using XLSTAT software, version 2019.2 (Addinsoft, Paris, France).

## 3. Results

**Figure 1:**
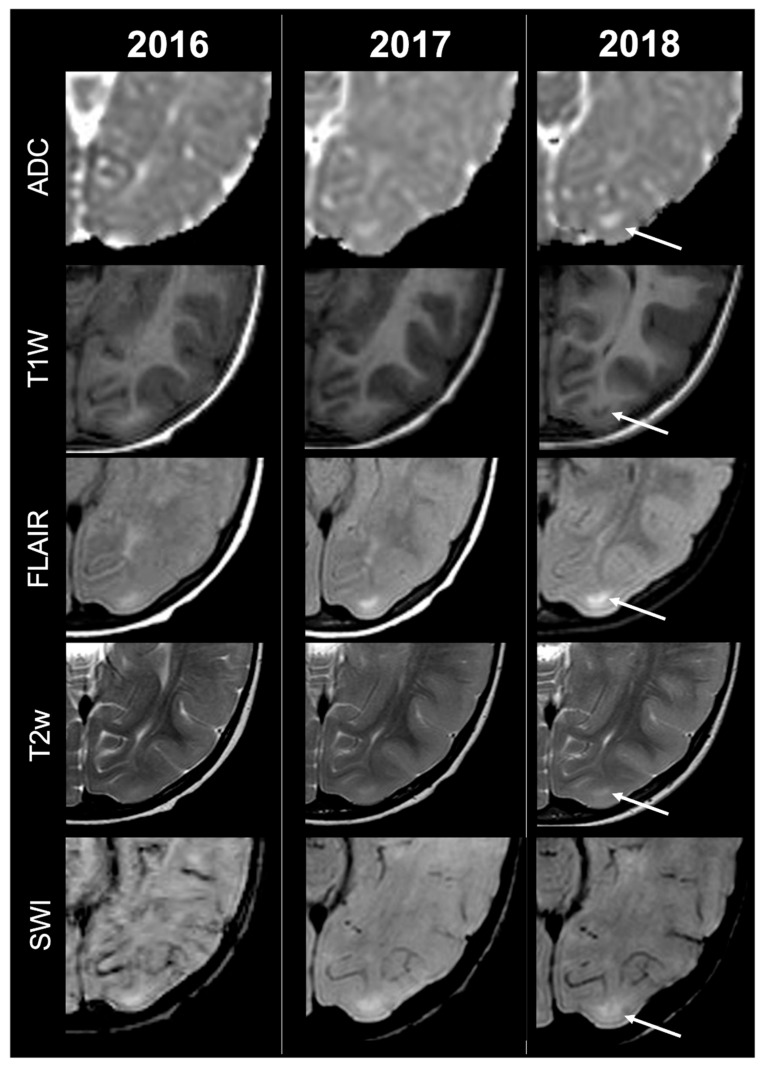
Left occipital tuber transition from type A to B at three different timepoints in a male patient diagnosed with TSC1.

**Figure 2:**
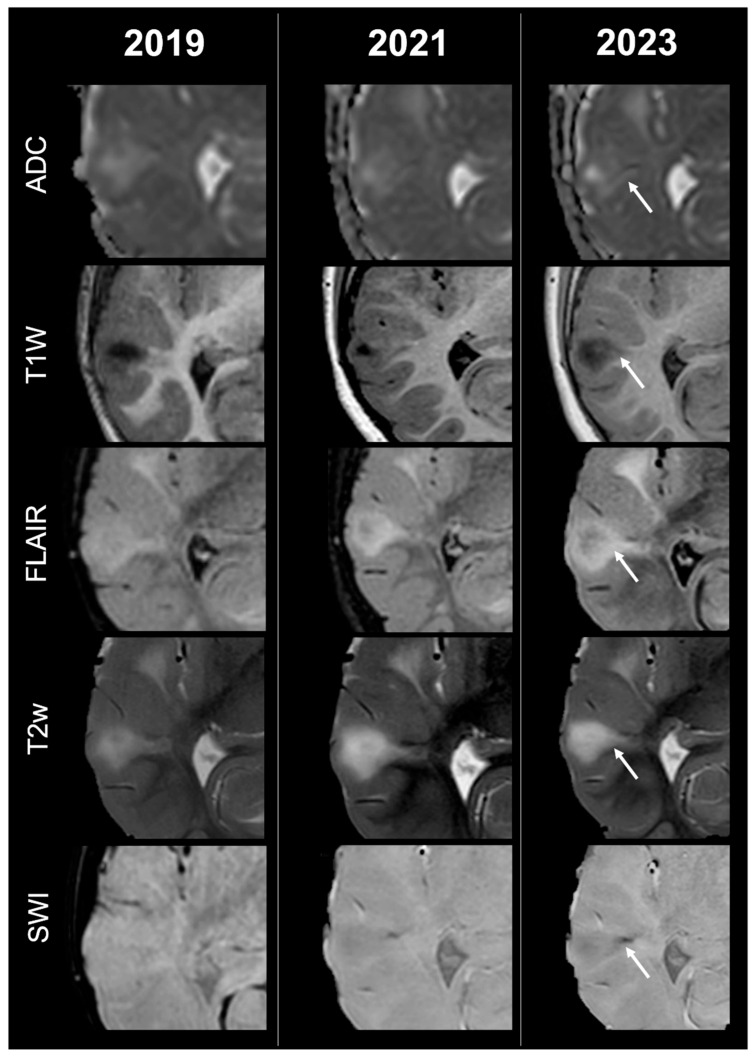
Right temporal tuber transition from type B to C1 at three different timepoints in a male patient diagnosed with TSC2.

**Figure 3:**
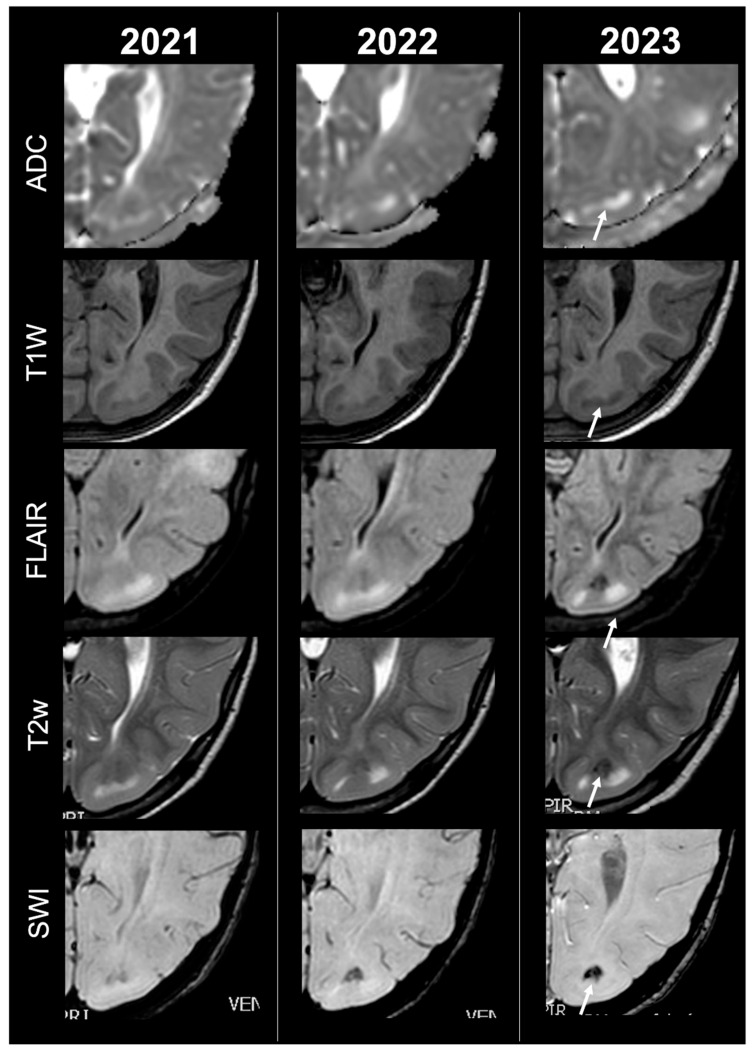
Left occipital tuber transition from type C1 to C2 at three different timepoints in a female patient diagnosed with TSC1.

**Figure 4:**
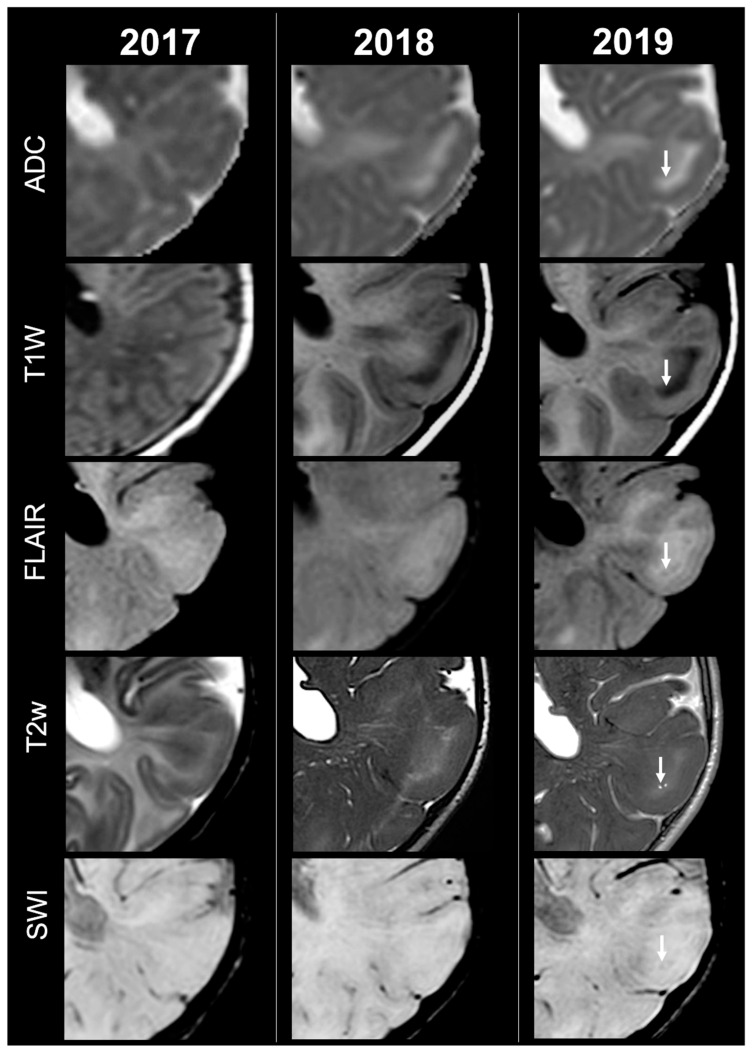
Left parietal tuber transition from type A to D at three different timepoints in a female patient diagnosed with TSC2.

Concerning total lesion count, the interobserver agreement was excellent, with an ICC of 0.91 (95% CI: 0.87–0.95). At baseline, the total number of CTs identified across the cohort was 918 (mean ± SD = 16.1 ± 12.4), whereas at the last available MRI follow-up, the overall count increased to 1070 (mean ± SD = 18.8 ± 13.3), corresponding to a net gain of 152 CTs, equal to a relative variation of +17%. When stratified by tuber type, distinct trajectories were observed over time. Type A CTs, which were relatively abundant at diagnosis (N = 303, mean ± SD = 5.3 ± 7.0), decreased to 255 (mean ± SD = 4.5 ± 5.9) at follow-up, yielding a reduction of 48 lesions (−16%). Conversely, type B CTs showed an opposite trend, increasing from 438 (mean ± SD = 7.7 ± 8.4) at baseline to 556 (mean ± SD = 9.8 ± 8.6) at follow-up, corresponding to a net increase of 118 tubers (+27%). An example of MRI signal evolution from tuber A to tuber B is shown in Figure 1. Similarly, type C1 CTs rose from 160 (mean ± SD = 2.8 ± 4.8) to 220 (mean ± SD = 3.9 ± 5.8), with a net gain of 60 tubers (+38%). Type C2 CTs, initially rare at diagnosis (N = 14, mean ± SD = 0.2 ± 0.8), markedly expanded to 33 (mean ± SD = 0.6 ± 1.5), accounting for an increase of 19 CTs (+136%). Finally, type D CTs were only sporadically encountered, with a modest increase from 3 (mean ± SD = 0.1 ± 0.3) to 6 (mean ± SD = 0.1 ± 0.4), equating to a 100% variation. The observed increment of type C2 tubers was attributable almost exclusively to the progressive transformation of pre-existing type B lesions and, more prominently, type C1 lesions, while the increase in type C1 tubers reflected the gradual conversion of both type A and type B CTs that were already detectable at disease onset. An example of MRI signal evolution from tuber B to C1 is shown in Figure 2, while from tuber C1 to C2 in Figure 3. Only in one patient was a type C1 tuber newly identified at follow-up without clear evidence at baseline. In contrast, newly apparent tubers emerging over time but not clearly visible on baseline scans were most frequently represented by type A and type B lesions. Type D tubers were rare overall and were identified in only six lesions from four patients with a particularly high lesion burden; in all but one case they represented the evolution of type A or type B CTs (an example of MRI signal evolution from tuber A to tuber D is shown in Figure 4), while in a single case they were associated with adjacent subtle calcific foci. Overall descriptive data are summarized in Table 1.

To evaluate whether the observed longitudinal changes were statistically significant, a paired Student’s *t*-test comparing baseline and follow-up tuber counts was performed. This analysis confirmed a significant increase over time: the mean number of CTs rose from 16.11 ± 12.43 at baseline to 18.77 ± 13.29 at follow-up, yielding a mean paired difference of −2.67 (95% CI [−3.94, −1.39]); the test statistic was t (56) = 4.19, corresponding to *p* < 0.0001, indicating a highly significant increase in overall tuber burden.

**Figure 5:**
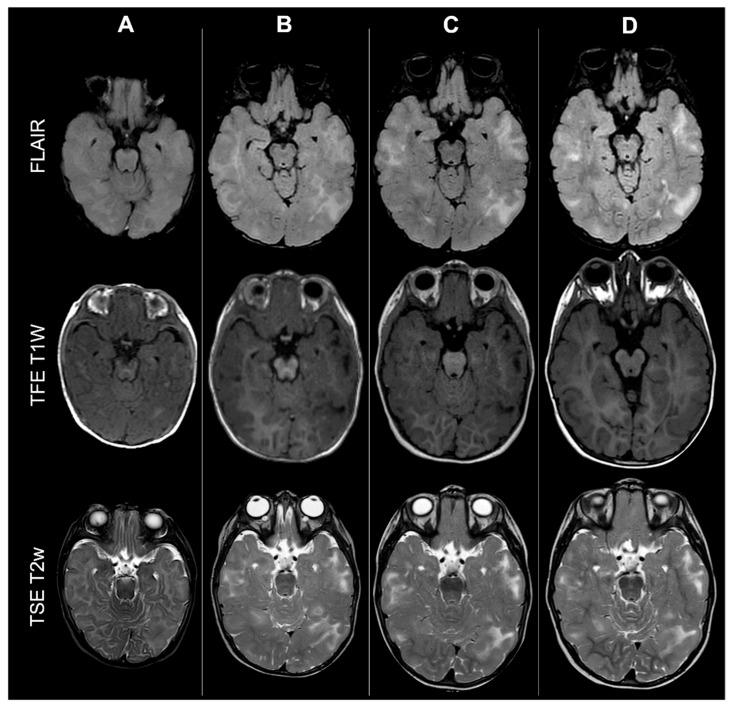
Effect of myelination on cerebral cortical tubers’ count: MRI scans from the same male TSC2 patient acquired at four different timepoints (respectively: 6 months old—column **A**; 18 months old—column **B**; 36 months old—column **C**; 4 years old—column **D**, showing how incomplete myelination in early infancy may impact tubers detection by reducing contrast with surrounding brain tissue.

Given the potential role of brain maturation in influencing CT detection and classification, patients were subsequently stratified into two age-defined subgroups according to myelination status (<2 years vs. ≥2 years at baseline). An example of the effect of incomplete myelination on cerebral CTs count based on MRI scan is shown in Figure 5.

In the subgroup aged <2 years, corresponding to patients with incomplete myelination, the total number of CTs at diagnosis was 467 (mean ± SD = 19.5 ± 15.2), increasing to 580 at follow-up (mean ± SD = 24.2 ± 15.8), with a net gain of 113 tubers (+24%). Subtype analysis demonstrated a heterogeneous distribution: type A CTs decreased from 214 (mean ± SD = 8.9 ± 8.7) to 163 (mean ± SD = 6.8 ± 7.3), representing a loss of 51 tubers (−24%); type B CTs increased from 157 (mean ± SD = 6.5 ± 8.8) to 247 (mean ± SD = 10.3 ± 9.2), with a net increase of 90 (+57%); type C1 CTs grew from 93 (mean ± SD = 3.9 ± 6.8) to 153 (mean ± SD = 6.4 ± 7.8), with a net increase of 60 (+65%); type C2 CTs rose dramatically from 1 (mean ± SD = 0.0 ± 0.2) to 12 (mean ± SD = 0.5 ± 1.0), equating to an increase of 11 CTs (+1100%); while type D CTs, though rare, rose from 2 (mean ± SD = 0.1 ± 0.4) to 5 (mean ± SD = 0.2 ± 0.6), corresponding to +3 tubers (+150%). Statistical analysis with paired *t*-testing confirmed the significance of these longitudinal changes: mean values increased from 19.46 ± 15.21 at baseline to 24.17 ± 15.75 at follow-up, with a mean difference of −4.71 (95% CI [−7.37, −2.04]); the test statistic was t (23) = 3.65, *p* = 0.0013, indicating a robust increase in tuber burden within this younger subgroup.

In the subgroup aged ≥2 years, corresponding to patients with completed myelination, the overall CT burden at diagnosis was lower, totaling 451 tubers (mean ± SD = 13.7 ± 9.4), which increased modestly to 490 (mean ± SD = 14.8 ± 9.6) at follow-up, equating to a net increase of 39 tubers (+9%). Subtype analysis revealed stable type A tubers, increasing slightly from 89 (mean ± SD = 2.7 ± 3.9) to 92 (mean ± SD = 2.8 ± 3.8), a net gain of 3 (+3%); type B CTs rose from 281 (mean ± SD = 8.5 ± 8.1) to 309 (mean ± SD = 9.4 ± 8.2), corresponding to +28 (+10%); type C1 CTs remained stable, with 67 tubers (mean ± SD = 2.0 ± 2.4) identified at both baseline and follow-up, indicating no change (0%); type C2 CTs increased from 13 (mean ± SD = 0.4 ± 1.0) to 21 (mean ± SD = 0.6 ± 1.7), corresponding to +8 (+62%); and type D CTs remained unchanged at 1 (mean ± SD = 0.0 ± 0.2). Despite the relatively modest increase compared with the younger subgroup, paired *t*-testing nonetheless revealed a significant rise in overall CT count: mean values increased from 13.67 ± 9.45 at baseline to 14.85 ± 9.64 at follow-up, with a mean difference of −1.18 (95% CI [−2.08, −0.28]); the test statistic was t (32) = 2.68, *p* = 0.0115, confirming a statistically significant but smaller increase relative to the <2 years subgroup.

**Figure 6:**
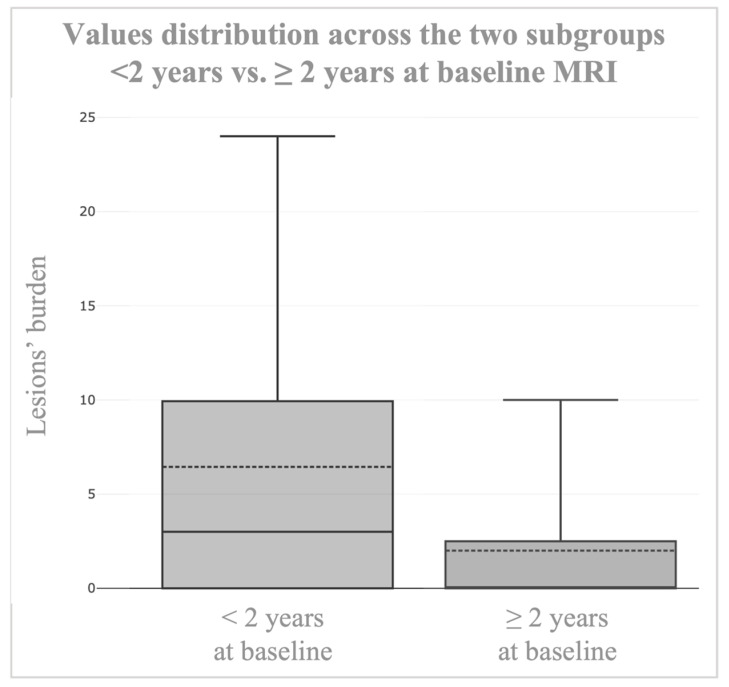
Values distribution across the two subgroups, namely <2 years vs. ≥2 years, at baseline MRI.

Finally, to directly compare the two age-based groups, a Welch’s two-sample *t*-test was applied, given the evidence of unequal variances. This analysis demonstrated that the mean CTs count in patients aged <2 years was significantly greater than in those aged ≥2 years, with a mean difference of 3.53 ± 1.36, test statistic T = 2.59, degrees of freedom = 28.4, and a right-tailed *p* value of 0.0075. The 95% confidence interval for the mean difference was 1.10, ∞. The corresponding effect size was large (Cohen’s d = 0.78), suggesting a clinically relevant difference between subgroups. As expected, the equality of variances assumption was not met (F-test, *p* < 0.00001), and both groups deviated significantly from normality on Shapiro–Wilk testing (*p* < 0.001); however, Welch’s *t*-test was chosen for its robustness to unequal variances and sample sizes, providing a reliable assessment of group differences even under deviations from normality. Outlier analysis, performed using the Tukey fence method (k = 1.5), identified 2 cases (8.3%) in the <2 years subgroup and 8 cases (24.2%) in the ≥2 years subgroup, although their removal did not alter the overall significance of the results. Values distribution across the two subgroups, namely <2 years vs. ≥2 years at baseline MRI, is shown in Figure 6.

## 4. Discussion

This retrospective study provides novel insights into the longitudinal evolution of CTs in pediatric patients diagnosed with TSC, highlighting how tuber burden and subtype distribution change over time in relation to age and myelination status.

Historically, CTs have been considered structurally stable lesions over time, that do not change in number or size beyond what is proportional to normal brain growth [29]. However, in recent times some sporadic evidence of calcific- and cyst-like changes within tuberal tissue have been described [23,25,27], and calcifications prevalence within CTs has been reported higher in older age groups of patients compared to younger ones [30,31,32], thus raising the issue whether and when inner changes occur over time. Few Authors also reported significant variations in tubers count within specific CNS regions [25,26], as well as microstructural longitudinal changes involving white matter, tuber and peri-tuberal tissues (detectable exclusively through advanced MRI acquisitions) [24]. Based on these preliminary data, we hypothesized that CTs do not behave as static but rather as dynamic lesions, both in terms of number and signal characteristics, regardless of the degree of neural tissue myelination or the patient’s age. Indeed, we found an overall statistically significant increase in CT number across the cohort. Importantly, tuber subtypes followed distinct trajectories: type A tubers tended to decrease in prevalence, whereas type B tubers increased, and more advanced morphologies—particularly calcified (C1 and C2) and cystic (D) forms—emerged over time. This pattern suggests a dynamic process in which early, relatively simple lesions undergo progressive structural transformation, and that pathological changes within the tuber leading to calcium deposition begin since a very early stages of cerebral development; this observation is even more important from a clinical perspective when considering that CTs with inner calcifications are considered to be more epileptogenic compared to smaller CTs without calcifications [33]. Finally, of note, type C2 tubers, although rare at baseline, exhibited the highest relative increase, supporting their role as markers of disease evolution and possibly of clinical severity [17,34].

A subsequent stratified analysis by age was performed to determine the extent to which myelination influences the detection and overall count of cerebral CTs, thereby potentially affecting the evaluation of total lesion burden and, consequently, the prediction of disease severity. Such analysis revealed that younger patients with clinical suspicion of TSC (<2 years at baseline) not only presented with a high initial CT burden (thus helping in confirming the initial MRI-based TSC diagnosis), but also demonstrated a more pronounced longitudinal increases in both total number and in the transition toward complex CTs’ subtypes. This finding underscores the impact of early brain maturation on lesion detectability and progression, and it corroborates the hypothesis that incomplete myelination at the time of baseline imaging may partly mask the presence of certain cerebral CT types, especially pure intracortical ones which later become more apparent as myelination advances [28,35]. By contrast, patients aged ≥2 years at baseline displayed a more modest though still statistically significant increase in CT count, indicating that disease progression is not limited to infancy but is more accentuated in early childhood; therefore, we could assume that the described increase in overall number accounts at least in part to an actual disease progression over time. However, to overcome any reasonable doubt about CTs progression further study are still needed, both increasing sample size, excluding infants from the analysis, and replicating the study on adults. This would give us a larger window of time to evaluate CTs evolution and validate the presented results.

From a clinical perspective, these results reinforce the importance of early and serial neuroimaging in TSC. Identifying patients with higher lesion burden and more advanced tuber morphologies at younger ages may facilitate stratification of risk for refractory epilepsy and poor neurodevelopmental outcome, ultimately supporting more tailored surveillance and management strategies. Because the number and conspicuity of such lesions in our cohort is relevant also in younger subjects, these findings support the notion that infant age does not affect the correct MRI-based diagnosis of TSC; therefore, according to current literature evidence [28,36], if TSC is clinically suspected since early infancy, neuroimaging should not be delayed (although careful longitudinal imaging follow-up is mandatory for an accurate assessment of the overall lesion burden and for a precise characterization of the location and size of cerebral CTs). Furthermore, the use of a refined MRI-based tuber classification offers a potentially valuable tool for future studies, as it accounts for calcification and cystic changes that have been underrepresented in previous classification systems, while also showing correspondence with the natural evolution of CTs.

Strengths of this study include the use of a homogeneous MRI protocol performed on the same scanner across all examinations (minimizing technical variability), the longitudinal design with repeated measures within the same patients, and the blinded consensus review by two experienced neuroradiologists (reducing inter-reader variability); in addition, the introduction of a novel CTs’ classification framework adds granularity to the assessment of disease progression. Nonetheless, several limitations should be acknowledged. The retrospective nature of the study imposes inherent constraints, including potential selection bias, as only term infants and patients with complete data and imaging follow-up could be included. Indeed, when referred to our tertiary hub from primary or secondary hospitals, patients with previous external MRI examinations—often lacking specific sequences such as SWI—were not subjected to repeat in-house imaging, particularly when sedation of the young patient would have been required; this inevitably resulted in a subset of “incomplete” examinations, which were therefore excluded from the present study. Regarding preterm subjects, their exclusion was motivated by the potential presence of confounding factors such as calcifications secondary to hemorrhage, brain injury related to hypoxia–ischemia, as well as venous sinus thrombosis, masses, or structural abnormalities. As for treatment-related confounders, we only included newly diagnosed cases without prior surgical or image-guided interventions. The sole exception was represented by patients who, during follow-up, underwent surgery for drug-resistant epilepsy (either cortical tuber resection or MRI-guided laser interstitial thermal therapy); in these few cases, the MRI performed for presurgical planning was considered the last available follow-up study, given its diagnostic completeness and clinical relevance. The modest sample size, though comparable to prior TSC cohorts, may limit generalizability, especially when stratifying by genetic mutation type or other clinical variables (an aim that goes far beyond the purposes of the present study). Moreover, another possible limitation of the study is that follow-up intervals varied across patients and the analysis was based on the raw difference between baseline and last examinations without normalization for follow-up duration. Statistical assumptions were occasionally challenged by non-normal distributions and unequal variances, though robust methods such as Welch’s *t*-test were applied to mitigate these issues. Furthermore, while our imaging protocol was consistent, the reliance on 1.5 T MRI may have limited sensitivity for very subtle cortical abnormalities compared with higher-field scanners; nevertheless, it should be acknowledged that this field strength represents the most widely employed standard for the neuroimaging evaluation of pediatric CNS disorders, thus consequently the present results may reasonably be regarded as representative of a real-world clinical setting. Finally, clinical correlates such as seizure severity, cognitive outcomes, or treatment history were not systematically analyzed in this work, and future prospective studies are needed to establish direct links between imaging progression and clinical course.

## 5. Conclusions

In conclusion, this study demonstrates that CTs in pediatric TSC are dynamic lesions, rather than static, evolving over time in both number and morphology. Younger patients, particularly those first examined in early infancy, are especially susceptible to increases in lesion burden and to progression toward calcified and cystic subtypes, supporting the hypothesis that age-dependent brain maturation and incomplete myelination contribute to the dynamic visibility and apparent growth of CTs across serial imaging. Importantly, our findings confirm that infant age does not compromise the accuracy of MRI-based diagnosis of TSC, underscoring that neuroimaging should not be delayed when clinical suspicion arises. Nevertheless, careful longitudinal MRI follow-up remains essential for comprehensive evaluation of lesion burden and precise characterization of tubers location and size. Furthermore, the refined MRI-based classification proposed here, incorporating features such as calcification and cystic changes that were underrepresented in earlier systems, provides a structured framework for future research. Collectively, these findings emphasize the critical interplay between neurodevelopmental stage and lesion detectability, strongly support age-specific imaging surveillance protocols, and lay the groundwork for integrating imaging biomarkers with clinical and genetic data to advance personalized, precision-based care for children with TSC.

## Figures and Tables

**Table 1:** Results from descriptive statistics on cortical tuber types’ MRI signal and number variation over time in the whole TSC patient cohort.

		N	%	MEAN	SD	MIN	MAX
**CTs at first MRI**	**Overall**	918	100%	16.1	12.4	0	53
	**Type A**	303	33%	5.3	7	0	25
	**Type B**	438	48%	7.7	8.4	0	31
	**Type C1**	160	17%	2.8	4.8	0	26
	**Type C2**	14	2%	0.2	0.8	0	5
	**Type D**	3	0%	0.1	0.3	0	2
**CTs at last MRI**	**Overall**	1070	100%	18.8	13.3	0	54
	**Type A**	255	24%	4.5	5.9	0	26
	**Type B**	556	52%	9.8	8.6	0	32
	**Type C1**	220	21%	3.9	5.8	0	26
	**Type C2**	33	3%	0.6	1.5	0	9
	**Type D**	6	1%	0.1	0.4	0	2
**CTs variation**	**Overall**	152	17%	2.7	4.8	0	24
	**Type A**	−48	−16%	−0.8	4.6	−23	6
	**Type B**	118	27%	2.1	6.3	−22	29
	**Type C1**	60	38%	1.1	3.9	−3	23
	**Type C2**	19	136%	0.3	0.9	0	4
	**Type D**	3	100%	0.1	0.3	0	2

Legend: N = number; SD = standard deviation; MIN = minimum number; MAX = maximum number; CT(s) = cortical tuber(s); MRI = magnetic resonance imaging.

## Data Availability

Data available on request due to restrictions (e.g., privacy and ethical reasons—minor patients).

## Abbreviations

ADC = apparent diffusion coefficient; CNS = central nervous system; CT = cortical tuber; DWI = diffusion-weighted imaging; FLAIR = fluid-attenuated inversion recovery; ICC = intraclass correlation coefficient; MRI = magnetic resonance imaging; SD = standard deviation; SWI = susceptibility-weighted imaging; TSC = tuberous sclerosis complex.
